# Cocaine in the Treatment of Whooping Cough

**Published:** 1886-02

**Authors:** 


### COCAINE IN THE TREATMENT OF WHOOPING COUGH.

In a pamphlet recently published, Dr. Moncorvo (*Lancet*, Sept. 6, 1885,) has advocated the employment of cocaine as an adjunct to the treatment of the malady by resorcin, for which Dr. Moncorvo stands the sponsor. The upper parts of the larynx are first to be mopped with the one per cent. solution of resorcin, and then a ten per cent. solution of the hydrochlorate of cocaine is applied. The cocaine should be used frequently.

